# Phase Angle: A Possible Biomarker to Quantify Inflammation in Subjects with Obesity and 25(OH)D Deficiency

**DOI:** 10.3390/nu11081747

**Published:** 2019-07-29

**Authors:** Luigi Barrea, Giovanna Muscogiuri, Daniela Laudisio, Carolina Di Somma, Ciro Salzano, Gabriella Pugliese, Giulia de Alteriis, Annamaria Colao, Silvia Savastano

**Affiliations:** 1 Dipartimento di Medicina Clinica e Chirurgia, Unit of Endocrinology, Federico II University Medical School of Naples, Via Sergio Pansini 5, 80131 Naples, Italy; 2 IRCCS, SDN, Via Gianturco 113, 80143 Naples, Italy

**Keywords:** Vitamin D, Obesity, Phase Angle (PhA), Bioelectrical Impedance Analysis (BIA), Inflammation

## Abstract

Obesity is associated to chronic low-grade metabolic inflammation and hypovitaminosis D. Among extra-skeletal effects, an important role in inflammation has been described for vitamin D (25(OH)D). Phase angle (PhA) is a bioelectrical impedance analysis (BIA) parameter that represents an indicator of cellular health in chronic inflammatory states. However, it is still unknown whether a low 25(OH)D levels might correlate with PhA in obesity. Considering the lack of evidence correlating the 25(OH)D levels with PhA in obesity, the aim of this study was to investigate their possible relationship in a group of patients with obesity stratified according to body mass index (BMI) categories. Four hundred and fifty-five adult subjects (219 males and 236 females; 36 ± 11 years) were enrolled. Body composition, including PhA, was assessed using a BIA phase-sensitive system. Serum levels of 25(OH)D was determined by a direct competitive chemiluminescence immunoassay. Most of the participants were affected by grade III obesity (24%) and had 25(OH)D deficiency (67%). Subjects with 25(OH)D deficiency had highest BMI (*p* < 0.001). Stratifying the sample population according to the BMI classes, 25(OH)D levels decreased significantly along with the increase in BMI (*p* < 0.001), with the lowest 25(OH)D levels in the class III obesity. In addition, stratifying the sample population according to 25(OH)D categories, BMI and fat mass (FM) decreased, while PhA increased significantly along with the 25(OH)D categories (*p* < 0.001). The 25(OH)D levels showed significant positive associations with PhA (*r* = −0.59, *p* < 0.001), and this association remained significant also after adjusting for BMI and FM (*r* = 0.60, *p* < 0.001). The lowest values of PhA were significantly associated with the severity of obesity (OR 0.3, *p* < 0.001) and of 25(OH)D deficiency (OR 0.2, *p* < 0.001). To compare the relative predictive power of body composition parameters associated with the 25(OH)D levels, we performed a multiple linear regression analysis. The most sensitive and specific cut-off for 25(OH)D levels to predict the PhA above the median was >14 ng/mL (*p* < 0.001). In conclusion, we provided preliminary insights into a novel link between 25(OH)D levels and PhA in the setting of obesity. This association uncovered a new potential usefulness of PhA as expression of cell membrane integrity and predictor of inflammation in low 25(OH)D status that might help in identifying high-risk patients with obesity who could benefit from careful 25(OH)D supplementation.

## 1. Introduction

Vitamin D is a fat-soluble vitamin which can be introduced with diet, but is mainly produced endogenously in the skin by conversion from 7-dehydrocholesterol upon ultraviolet-B radiation [1]. The major circulating form of vitamin D is the 25(OH)D and its serum concentration is considered a gold standard biomarker to assess vitamin D status in humans [2]. Observational studies as well as meta-analysis suggested a link between low 25(OH)D status and metabolic disorders, such as obesity [3,4,5]. Low 25(OH)D status and obesity, whose prevalence is increasing rapidly worldwide [6,7], are both involved in the development of chronic diseases, including heart disease, stroke, some types of cancer, chronic respiratory diseases, sleep disturbance, neurological [8,9,10,11,12,13], and inflammatory diseases [14,15,16], thus significantly increasing national health service costs [17]. 

Obesity, which is characterized by hypertrophy or/and hyperplasia of adipose tissue, contributes to "meta-inflammation" [18], a phenomenon characterized by chronic low-grade metabolic inflammation [19] that acts as a key underlying mechanism for the development of obesity-related diseases [20]. The meta-inflammation is characterized by increased levels of inflammatory mediators, which may induce cellular damage and cell death through apoptosis or necrosis [21]. However, monitoring these markers in subjects with obesity might require expensive biochemical analysis, with limited use in clinical practice. The expression of vitamin D receptors and vitamin D metabolizing enzymes in adipocytes supports the hypothesis of anti-adipogenic activity of vitamin D [22,23]. In addition, the expression of vitamin D receptors on inflammatory cells, including adipose tissue resident immune cells, suggested that there is a potential role for vitamin D also in the regulation of cytokine release and adipose tissue inflammation mediated by the inhibition of NF-κB signalling [24]. 

Bioelectrical impedance analysis (BIA) is a technique commonly used in a clinical setting for assessing body composition and to estimate fat and lean body mass. In addition, BIA provides the value of the phase angle (PhA) [25]. PhA has been widely considered a marker of inflammatory diseases [26,27]. Consequently, on the one hand, PhA is frequently lower than normal and its use has been recommended as a prognostic marker of morbidity and mortality in various chronic inflammatory states [28], including obesity [29]. On the other hand, the PhA increases along with the improvement of the clinical status [30]. In this context, as PhA represents an indicator of cellular health [31,32], BIA may provide an easy approach to identify cellular damage and cell death in chronic inflammatory states [33]. However, it is still unknown whether a low 25(OH)D status might represent a predictor of low PhA in patients with obesity. Considering the lack of evidence correlating the 25(OH)D levels with PhA in obesity, the aim of this study was to investigate their possible relationship in a group of patients with obesity stratified according to body mass index (BMI) categories.

## 2. Materials and Methods 

### 2.1. Design and Setting

This is a cross-sectional observational study carried out at the Department of Clinical Medicine and Surgery, Unit of Endocrinology, University Federico II, Naples (Italy), from October 2016 to March 2019. The work has been carried out in accordance with the Code of Ethics of the World Medical Association (Declaration of Helsinki) for experiments involving humans, and it has been approved by the Ethical Committee of the University of Naples “Federico II” Medical School (n. 173/16). The aim of the protocol was explained to all the study participants that signed written informed consents. 

### 2.2. Population Study

**Figure 1:**
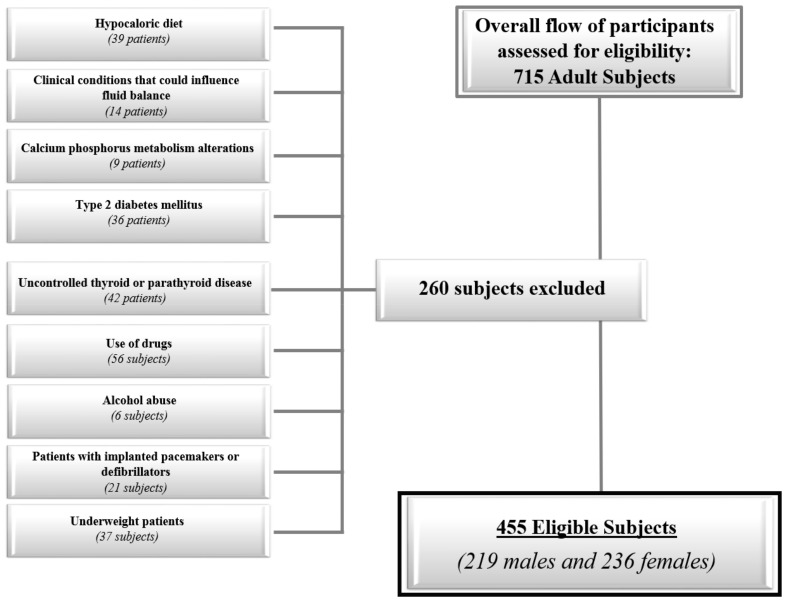
The flow chart of the study subjects.

Recruitment strategies included a sample of 455 of adult Caucasians subjects (18–57 years) of both genders consecutively enrolled among patients in our outpatient clinic, hospital volunteers, and employees, and resided in the Naples metropolitan area (latitude 40°49′N; elevation 17 m). The subjects were evaluated from November through March 2017 and November through March 2018. All female subjects were non-pregnant and non-lactating, and were evaluated in the follicular phase of the menstrual cycle. A full medical history, including drug use, was collected. In order to increase the homogeneity of the subject samples, we included only adults of both genders with the following criteria of exclusion:

- Hypocaloric diet in the last three months (39 subjects);
- Clinical conditions that could influence fluid balance, including liver or renal failure, cancer, and acute or chronic inflammatory diseases, based on a complete medical examination and laboratory investigations (14 subjects);
- Altered levels of serum creatinine, serum calcium, or albumin (9 subjects);
- Presence of type 2 diabetes mellitus (T2DM) (defined by criteria of the American Diabetes Association as follows: Basal plasma glucose level ≥126 mg/dL on two occasions, or glycated haemoglobin (HbA1c) ≥6.5% (≥48 mmol/moL) on two occasions, or both at the same time. Participants on antidiabetic medication, were considered to have T2DM (36 subjects);
- Uncontrolled thyroid or parathyroid disease (42 subjects);
- Current therapy with calcium, vitamin D supplementation, or osteoporosis therapies, anti-inflammatory drugs, statin, and other hypolipidemic agents (56 subjects);
- Alcohol abuse according to the Diagnostic and Statistical Manual of Mental Disorders (DSM)-V diagnostic criteria (6 subjects);
- Patients with implanted pacemakers or defibrillators because of the theoretical possibility of interference with the device activity due to the field of current induced by the impedance measurements (21 subjects);
- Underweight patients with BMI <18.5 kg/m^2^ (29 females and 8 males). The flow chart of study subjects is shown in Figure 1.

### 2.3. Power Justification

The power sample was calculated by the differences of means + standard deviation (SD) of PhA in the study population based on 25(OH)D categories (normal vs. deficiency/insufficiency). In particular, this difference was 5.7 ± 0.7 vs. 6.8 ± 0.9, *p* < 0.001). Considering a number of cases required in each group were set at 17 subjects, a type I (alpha) error of 0.01 (95%), and a type II (beta) of 0.05, the calculated power size was 95%. The calculation of power size was performed using Sample Size Calculator Clinical Calc (https://clincalc.com/stats/samplesize.aspx).

### 2.4. Lifestyle Habits

We defined current smokers (smoking at least one cigarette per day), or non-current smokers; (YES/NO). Subjects were defined as physically active if habitually engaged in at least 30 min/day of physical aerobic exercise; (YES/NO); as previously reported [34,35,36,37].

### 2.5. Anthropometric Measurements 

Measurements were performed in the morning, after an overnight fast. The measurements were made in a standard way by the same operator (a nutritionist experienced in providing nutritional assessment and body composition). At the beginning of the study, all anthropometric measurements were taken with subjects wearing only light clothes and without shoes, as previously reported [38,39,40,41]. In each subject, weight and height were measured to calculate the BMI (weight (kg) divided by height squared (m^2^), kg/m^2^). Height was measured to the nearest 0.5 cm using a wall-mounted stadiometer (Seca 711; Seca, Hamburg, Germany). Body weight was determined to the nearest 0.1 kg using a calibrated balance beam scale (Seca 711; Seca, Hamburg, Germany). BMI was classified according to World Health Organization’s criteria for normal weight: 18.5–24.9 kg/m^2^; overweight, 25.0–29.9 kg/m^2^; grade I obesity, 30.0–34.9 kg/m^2^; grade II obesity, 35.0–39.9 kg/m^2^; grade III obesity ≥40.0 kg/m^2^ [42]. 

### 2.6. Bioelectrical Impedance Analysis

Body composition was assessed using a BIA phase-sensitive system by experienced observers (an 800-µA current at a frequency single-frequency of 50 kilohertz (kHz) BIA 101 RJL, Akern Bioresearch, Florence, Italy) [43], as previously reported [44,45,46,47]. The exam was performed as suggested by the European Society of Parental and Enteral Nutrition (ESPEN) [32]. Electrodes (BIATRODES Akern Srl; Florence, Italy) were placed on the hand and the ipsilateral foot, according to Kushner (1992) [48]. All measurements were performed under strictly standardized conditions by a single nutritionist, using the same device in order to avoid interobserver and interdevice variability. The instrument was routinely checked with resistors and capacitors of known values. Reliability for within-day and between-day measurements by the same observer were <1.9% for resistance (R), <2.1% for reactance (Xc), and <2.8% for R, <2.5% for Xc, respectively. The coefficients of variation (CVs) of repeated measurements of R and Xc at 50 kHz was assessed in 12 individuals (6 males and 6 females) by the same observer: CVs were 1.6% for R and 1.4% for Xc. The PhA was derived from conditions under 50 kHz according to the following formula: PhA (°, degrees) = arctangent Xc/R ((Xc/R) × (180/π)).

### 2.7. Assay Methods

Samples were collected in the morning between 8 a.m. and 10 a.m., after an overnight fast of at least 8 h and stored at −80 °C until being processed. The 25(OH)D levels were quantified by a direct competitive chemiluminescence immunoassay (CLIA) (Liaison^®^, DiaSorin, Saluggia, Italy), with a specificity of 100% for 25(OH)D. The analytical measurement range of detection is 4–150 ng/mL, whereas the intra-assay CVs were 5.4%, 2.8%, and 4.7 % and the inter-assay CVs were 10.1%, 4.8%, and 7.9 % for low, medium, and high points of the standard curve, respectively; as previously reported [41,49]. The 25(OH)D deficiency was defined as a 25(OH)D levels <20 ng/mL (50 nmol/L), insufficiency between 21 and 29 ng/mL (from 52.5 to 72.5 nmol/L), and normal levels ≥ 30 ng/mL (75 nmol/L) [50]. 

### 2.8. Statistical Analysis

The data distribution was evaluated by Kolmogorov–Smirnov test and the abnormal data were normalized by logarithm. Skewed variables were back-transformed for presentation in tables and figures. Results are expressed as mean ± standard deviation (SD). The differences among the BMI classes and 25(OH)D categories, were analyzed by ANOVA followed by the Bonferroni post-hoc test. The correlations between study variables were performed using Pearson *r* correlation coefficients were estimated after adjusting for BMI and fat mass (FM). Proportional odds ratio (OR) models, 95% interval confidence (IC), and R^2^, were performed to assess the association among BMI classes and 25(OH)D categories. In addition, two multiple linear regression analysis models (stepwise method), expressed as R^2^, Beta (β) and *t*, with PhA as dependent variable were used to estimate the predictive value of: BMI and body composition parameters (Model 1) and with 25(OH)D levels as dependent variables were used to estimate the predictive value of: BMI and body composition parameters (Model 2). Receiver operator characteristic (ROC) curve analysis was performed to determine sensitivity and specificity, area under the curve (AUC), and IC, as well as cut-off value of 25(OH)D levels in detecting PhA. Test AUC for ROC analysis was also calculated and we entered 0.831 for AUC ROC and 0.5 for null hypothesis values. An α level of 0.05 (type 1 error) and a β level of 0.20 (type II error) were used as the cut-off values for statistical significance. Only variables that had a *p*-value <0.05 in the univariate analysis (partial correlation) were entered. Variables with a variance inflation factor (VIF) >10 were not considered to avoid multicollinearity. Values ≤5% were considered statistically significant. Data were collected and analyzed using the MedCalc^®^ package (Version 12.3.0 1993–2012, Mariakerke, Belgium). 

## 3. Results

Study population consisted of 455 participants; 219 males and 236 females. Age, anthropometric characteristics, and 25(OH)D levels of the study population are shown in Table 1. Most of the participants were grade III obesity (24.2%) and presented 25(OH)D deficiency (67.3%). The demographics information, including age, smoking habits, and physical activity for the different obesity categories, have been reported in the Table S1.

**Figure 2:**
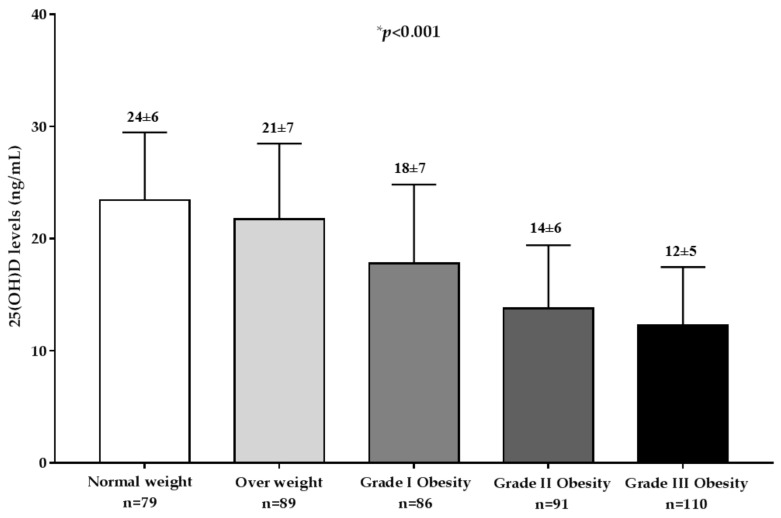
The 25(OH)D levels in the population study across body mass index (BMI) categories. A * *p* value denotes a significant difference (*p* < 0.05).

Figure 2 shows 25(OH)D levels in the population study across BMI categories. As reported, significant differences were observed in 25(OH)D levels. Stratifying the sample population according to the BMI classes, 25(OH)D levels decreased significantly along with the increase of BMI (*p* < 0.001).

All subjects completed the study protocol including BIA measurements. The difference of PhA across all BMI categories was reported in Figure S1. The PhA tended to increase with increasing BMI up to overweight, while decreasing in higher BMI groups. 

In Table 2 reported all body composition parameters (free fat mass, FFM; total body water, TBW; extra-cellular water, ECW; and intra-cellular water, ICW) of the study population. 

**Figure 3:**
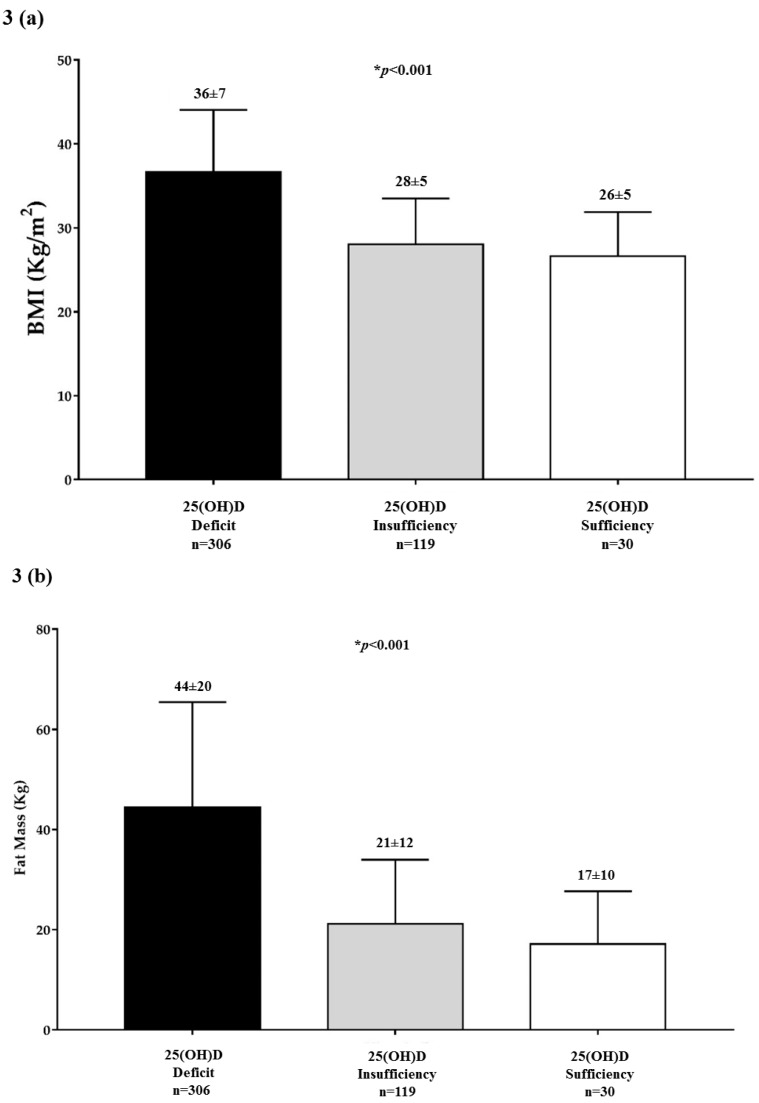

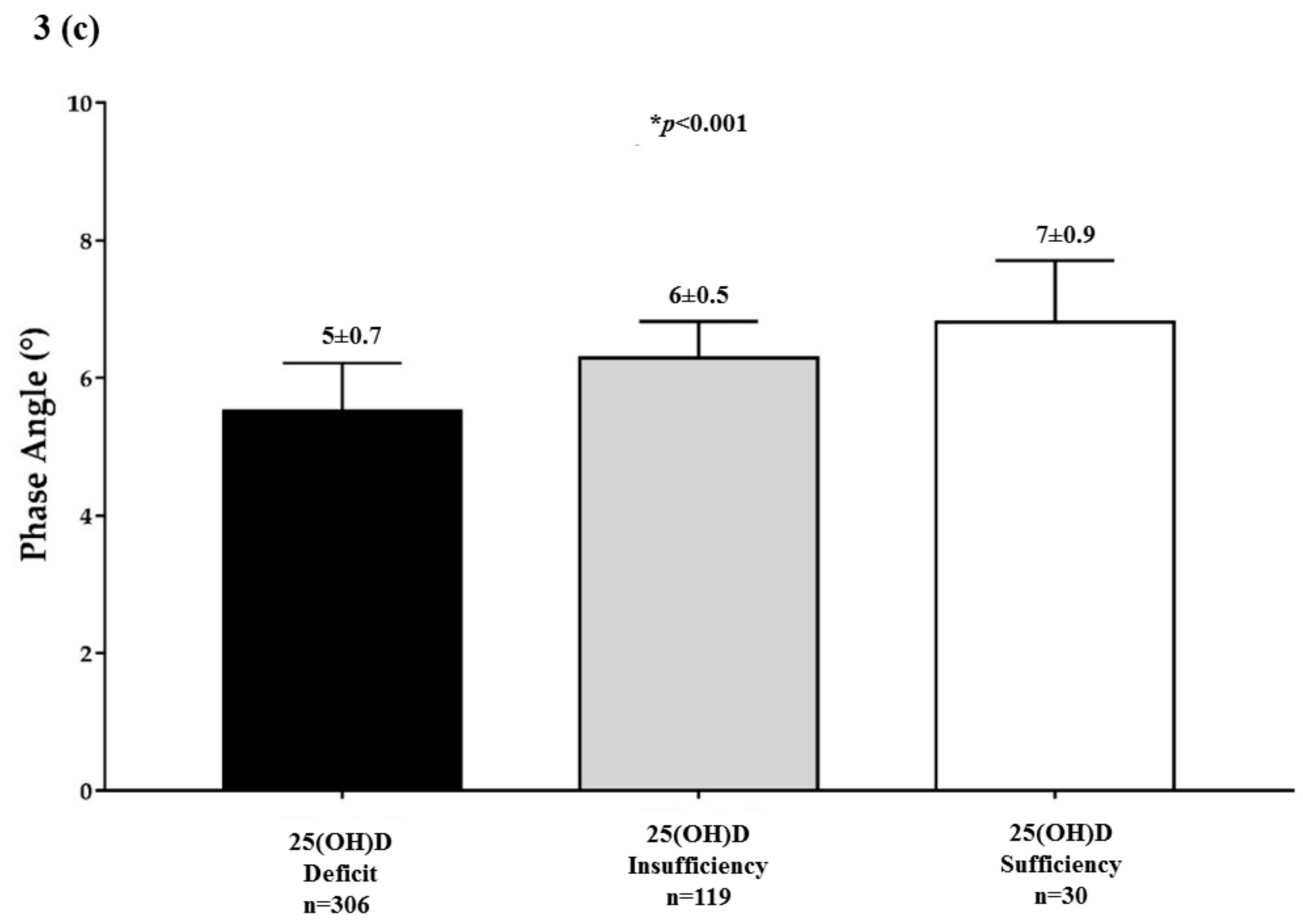
The body mass index (BMI) (Figure 3a), fat mass (FM) (Figure 3b) and phase angle (PhA) (Figure 3c) in the population study across 25(OH)D categories. A * *p* value denotes a significant difference (*p* < 0.05).

Figure 3 reported BMI (Figure 3a), FM (Figure 3b), and PhA (Figure 3c) in the population study across 25(OH)D categories. Stratifying the sample population according to 25(OH)D categories, BMI and FM decreased, while PhA increased significantly along with the 25(OH)D categories (*p* < 0.001).

### Correlation Analysis

**Figure 4:**
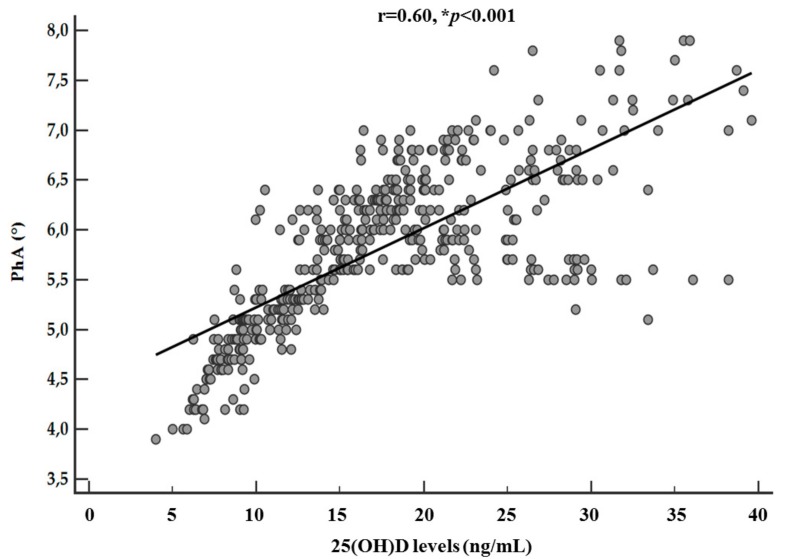
Correlation between 25(OH)D levels and PhA after adjusting for BMI and FM. A * *p* value denotes a significant difference (*p* < 0.05).

The correlations among 25(OH)D levels, age, anthropometric measurements, and body composition parameters have been summarized in Table 3. Apart from age, 25(OH)D levels showed significant associations with all parameters. In particular, the correlation between 25(OH)D levels and PhA remained significant also after adjusting for BMI and FM (r = 0.60, *p* < 0.001), as shown in Figure 4.

The results of the bivariate proportional OR model performed to assess the association of PhA and FM with quantitative variables were reported in Table 4. The lowest values of PhA and highest values of FM were significantly associated with the severity of obesity (OR 0.3, *p* < 0.001 and OR 1.3, *p* < 0.001; respectively) and deficit of 25(OH)D (OR 0.2, *p* < 0.001 and OR 1.1, *p* < 0.001; respectively). 

To compare the relative predictive power of BMI, age, and sex associated with PhA, we performed a multiple linear regression analysis model using BMI, age, and sex as independent variables and PhA as dependent variable. Using this model, BMI entered at the first step (*p* < 0.001) and explained 54% of PhA variability. Results were shown in Table 5.

To compare the relative predictive power of body composition parameters associated with the 25(OH)D levels, we performed a second multiple linear regression analysis using a model that included BMI, R, Xc, PhA, FM, FFM, ECW, and ICW as independent variables. Using this model, PhA entered at the first step (*p* < 0.001), while BMI, R, Xc, FM, FFM, ECW, and ICW were excluded. Results were reported in Table 6. 

**Figure 5:**
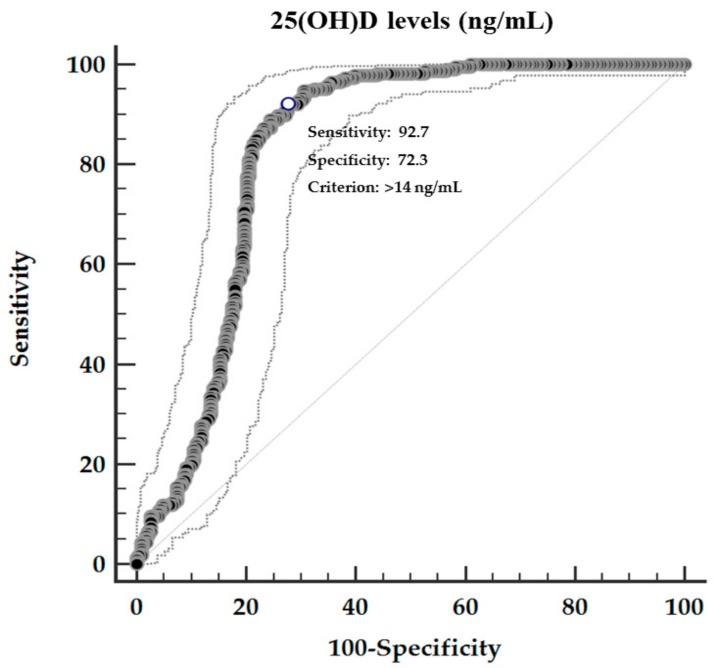
ROC for predictive values of 25(OH)D levels in detecting PhA.

A ROC analysis was then performed to determine the cut off value of 25(OH)D levels predictive of PhA. In particular, 25(OH)D levels >14 ng/mL (*p* < 0.001, AUC 0.83, standard error 0.02, 95% CI 0.8 to 0.9; Figure 5), could serve as threshold for significantly increased PhAs above the median (5.9°).

## 4. Discussion

In this cross-sectional, observational study we reported a novel direct association between 25(OH)D levels and PhA in a sample of an obese adult population stratified according to BMI categories. The stratification of the study population according to the BMI classes allowed us to confirm the negative relationship between 25(OH)D levels and body weight, being the lowest 25(OH)D levels present in study participants with class III BMI. When considering the entire sample population, lower 25(OH)D levels were observed and were associated not only with higher BMI but also with higher FM, as expected. In addition, our findings demonstrated that lower 25(OH)D levels were also associated to a lower PhA. In agreement with previous studies [26,27,31], low PhAs were significantly associated with the severity of obesity. Of interest, the association between 25(OH)D levels and PhA remained significant after adjustment for BMI and FM. At the multiple regression analysis, 25(OH)D levels were the major determinant of PhA while BMI and other BIA parameters were excluded. Finally, based on the ROC curve analysis, the most sensitive and specific cut-off for the 25(OH)D levels to predict the highest PhA were >14 ng/mL. To date, this is the first study investigating the association between 25(OH)D levels and PhA in patients with obesity.

Several studies indicated that 25(OH)D levels are lower in patients with obesity than in normal weight subjects [51,52]. A recent meta-analysis of randomized controlled trials (RCTs) showed that 25(OH)D levels were also inversely associated with the FM [53]. In fact, it is well known that BMI is only a measure of the total adiposity, without discriminating body fat amount and distribution [6]. Beyond its anti-adipogenic effect [22,54], the presence of vitamin D receptors and vitamin D metabolizing enzymes on inflammatory cells suggests the potential role for vitamin D in regulating inflammatory pathways [24]. In fact, it has been consistently reported that low 25(OH)D levels are accompanied by increased inflammation and consequent development of metabolic and cardiovascular diseases [55,56]. In addition, vitamin D acts as an immune-suppressor of inflammatory markers, including tumor necrosis factor-alfa (TNF-α) and interleukin (IL)-6, both of which are characteristically elevated in subjects with obesity [25,57]. Of interest, Amer et al. reported on more than 15,000 subjects from the National Health and Nutrition Examination Survey 2001–2006. The subjects were divided into low and high 25(OH)D levels with a cut-off of 21.2 ng/mL (53 nmol/L). In only subjects with a 25(OH)D levels <53 nmol/L, 25(OH)D levels was inversely associated with C-reactive protein (CRP) levels (*p* < 0.001), whereas there was no significant association in those with values greater than 53 nmol/L (*p* = 0.07) [58]. Therefore, translating these results to our study, the category of subjects with 25(OH)D insufficiency is just the category that can better benefit from 25(OH)D supplementation in the clinical setting of the obesity-related low-grade inflammation.

PhA is a BIA-derived raw parameter obtained from the arctangent of the Xc to R ratio that has been used as an indicator of cell membrane function [26] and inflammation [27,59]. To date, the clear biological significance of PhA has to be clarified. However, a growing body of evidence showed the reliability of PhA not only as an indicator of health status in various clinical settings, including cancer and psoriasis [26,33,60,61,62], but also as a tool to determine prognostic, nutritional, and cell membrane function values [63,64]. PhA reflects the cell membrane integrity and size, and the water distribution between the intracellular and extracellular water compartments. In particular, a low PhA indicates cell death or decreased cell integrity, whereas higher PhA is expression of larger quantities of intact cell membranes [26]. It has been well recognized that inflammation impairs PhA [26,65] and a significant relationship between lower PhA and inflammatory biomarkers has been described, including TNF-α, IL-6, and CRP levels [26].

However, similarly to 25(OH)D levels, PhA is related to body weight and body composition [27,31], especially FM [66,67]. In particular, in healthy populations, BMI is one of the major determinant of PhA [26,68]. We have also previously reported that, in a sample of adult population of both sexes evaluated on the basis of their adherence to the Mediterranean diet, subjects in the lowest tertile of PhA values had a higher BMI and showed the lower adherence to the Mediterranean diet [47]. This inverse correlation was confirmed also in the present study. Of interest, we found that the direct relationship between 25(OH)D levels and PhA remained significant after adjustment for both BMI and FM. In addition, among BMI and other BIA parameters, 25(OH)D levels was the major determinant of the PhA variability. This finding let us to hypothesize that 25(OH)D deficiency–related meta-inflammation in patients with obesity might be involved in alterations in cell membrane integrity and/or in intra/extra fluid balance, of which low PhA values might represent a sensitive biomarker. 

Limitations of this study warrant some considerations. The cross-sectional nature of the study did not allow to clearly determine the prognostic value of 25(OH)D levels for predicting PhA. Moreover, the suggested cut-off value of 25(OH)D levels for identifying high PhAs should be viewed with caution until results of studies in larger patient populations have become available to perform an appropriate cross-validation. Second, the possible underlying inflammatory status linking 25(OH)D levels and PhA should be better investigated measuring also serum inflammatory biomarkers such as TNF-α, IL-6, and CRP levels. Nevertheless, a major strength of this study includes the large sample of patients and the stratification of population in different BMI categories, making it possible the comparisons across subjects independently of BMI. Finally, our study was based on a single clinical center, with a possibility of selection bias on the results. Nonetheless, the single-center study allowed to increase the homogeneity of the sample as we included participants living in the same geographical area, with the same effect of latitude on vitamin D levels and, likely, with similar nutrient availability and food consumption patterns. 

In conclusion, our study provided preliminary insights into a novel link between 25(OH)D levels and PhA in an obesity setting. This association uncovered a new potential usefulness of PhA as expression of cell membrane integrity and predictor of inflammation in low 25(OH)D status. As possible translational applications, these findings: (i) Suggest that the determination of a specific cut-off value for the 25(OH)D levels might help in identifying high-risk patients with obesity with low PhAs, as biomarker of inflammation who could benefit from careful 25(OH)D supplementation; (ii) recommend the assessment of PhA as good clinical practice in the management of patients with obesity and low 25(OH)D status at high risk of low-grade inflammation. Further intervention clinical trials on large series populations will be of paramount importance to elucidate the potential translational application of the results of this study and the beneficial effects of the supplementation of 25(OH)D on the PhA as a sensitive biomarker of 25(OH)D deficiency-related inflammation in obesity.

## Figures and Tables

**Table 1:** Gender, lifestyle habits, age, anthropometric characteristics, and 25(OH)D levels of the study population.

Parameters	Mean ± SD or Number (%) *n* = 455
Gender	
Males	219 (48.1%)
Females	236 (51.9%)
Smoking	
Yes	146 (32.0%)
No	310 (68.0%)
Physical Activity	
Yes	118 (25.9%)
No	338 (74.1%)
Age (years)	37 ± 11
Weight (kg)	97± 25
Height (m)	1.69 ± 0.09
BMI (kg/m^2^)	34 ± 8
Normal weight	79, 17.4%
Over weight	89, 19.6%
Grade I obesity	86, 18.9%
Grade II obesity	91, 20.0%
Grade III obesity	110, 24.2%
25(OH)D levels (ng/mL)	17 ± 7.5
Deficiency	306, 67.3%
Insufficiency	119, 26.2%
Normal	30, 6.6%

**Table 2:** Body composition parameters of the study population.

Parameters	Mean ± SD *n* = 455
R (Ω)	475.6 ± 89.5
Xc (Ω)	48.1 ± 9.9
PhA (°)	5.8 ± 0.8
FM (kg)	36.6 ± 21.8
FM (%)	34.8 ± 14.4
FFM (kg)	60.9 ± 10.5
FFM (%)	65.2 ± 14.4
TBW (Lt)	45.3 ± 8.2
ECW (Lt)	21.2 ± 4.0
ICW (Lt)	24.1 ± 5.0

**Table 3:** Correlations among 25(OH)D levels, age, anthropometric measurements, and body composition parameters.

Parameters	25(OH)D Levels (ng/mL)	
	r	*p*-Value
Age (years)	−0.08	0.08
BMI (kg/m^2^)	−0.61	<0.001 **
R (Ω)	−0.18	<0.001 **
Xc (Ω)	0.34	<0.001 **
PhA (°)	0.74	<0.001 **
FM (kg)	−0.62	<0.001 **
FM (%)	−0.61	<0.001 **
FFM (kg)	−0.02	0.69
FFM (%)	0.61	<0.001 **
TBW (Lt)	−0.06	0.19
ECW (Lt)	−0.39	<0.001 **
ICW (Lt)	0.21	<0.001 **

A *p* value in bold type denotes a significant difference (** *p* < 0.05).

**Table 4:** Bivariate proportional odds ratio model to assess the association among PhA and FM, respectively, with BMI and 25(OH)D categories.

Parameters	PhA (°)				FM (kg)			
	OR	*p*-Value	95% IC	R^2^	OR	*p*-Value	95% IC	R^2^
**BMI**								
Normal weight	1.5	0.01 **	1.1–2.1	0.02	0.7	<0.001 **	0.7–0.8	0.43
Overweight	8.9	<0.001 **	5.5–14.7	0.24	0.9	<0.001 **	0.9–1.0	0.17
Grade I obesity	1.1	0.01 **	0.9–1.5	0.002	1.0	0.04 **	1.0–1.0	0.01
Grade II obesity	0.5	<0.001 **	0.4–0.7	0.05	1.0	<0.001 **	1.0–1.0	0.03
Grade III obesity	0.3	<0.001 **	0.2–0.4	0.15	1.3	<0.001 **	0.2–1.3	0.54
**25(OH)D levels**								
Deficit	0.2	<0.001 **	0.1–0.2	0.26	1.1	<0.001 **	1.1–1.1	0.29
Insufficiency	3.2	<0.001 **	2.3–4.5	0.13	0.9	<0.001 **	0.9–1.0	0.19
Sufficiency	8.0	<0.001 **	4.1–15.6	0.11	0.9	<0.001 **	0.9–1.0	0.07

A ** *p* value denotes a significant difference (*p* < 0.05).

**Table 5:** Multiple regression analysis models (stepwise method) with PhA as dependent variable to estimate the predictive value of: BMI, sex, and age.

Parameters	Multiple Regression Analysis			
*Model 1*	R^2^	β	t	*p* Value
**BMI**	0.54	−0.54	−14.3	<0.001 **
**Sex**	0.63	−0.32	−9.0	<0.001 **
**Age**	0.64	−0.11	−2.9	0.004 **

A ** *p* value denotes a significant difference (*p* < 0.05).

**Table 6:** Multiple regression analysis models (stepwise method) with the 25(OH)D levels as dependent variable to estimate the predictive value of: BMI and body composition parameters.

Parameters	Multiple Regression Analysis			
*Model 2*	R^2^	β	t	*p* Value
**PhA (°)**	0.55	0.74	23.6	<0.001 **
*Variables excluded: R, Xc, FM, FFM, ECW, ICW*				

A ** *p* value denotes a significant difference (*p* < 0.05).

## Supplementary Materials

The following are available online at https://www.mdpi.com/2072-6643/11/8/1747/s1, Table S1: The demographics information, including age, smoking habits, and physical activity for the different obesity categories, in study population. Figure S1: The difference of phase angle across all body mass index categories.

## Abbreviations

NF-κB	Nuclear Factor kappa light chain enhancer of activated B cells
BIA	Bioelectrical Impedance Analysis
PhA	Phase Angle
BMI	Body Mass Index
PTH	Parathyroid Hormone
T2DM	Type 2 Diabetes
HbA1c	Glycated Haemoglobin
DSM	Diagnostic and Statistical Manual of Mental Disorders
WHO	World Health Organization
kHz	Kilohertz
ESPEN	European Society of Parental and Enteral Nutrition
R	Resistance
Xc	Reactance
CVs	Coefficients of Variation
CLIA	Chemiluminescence Immunoassay
SD	Standard Deviation
FM	Fat Mass
OR	Proportional Odds Ratio
IC	Interval Confidence
ROC	Receiver Operator Characteristic
AUC	Area Under the Curve
FFM	Free Fat Mass
TBW	Total Body Water
ECW	Extra-cellular Water
ICW	Intra-cellular Water
RCTs	Randomized Controlled Trials
TNF-α	Tumor Necrosis Factor alfa
IL	Interleukin
CRP	C-Reactive Protein
