# Supplementary material for: Phase Angle: A Possible Biomarker to Quantify Inflammation in Subjects with Obesity and 25(OH)D Deficiency
Source: Nutrients. 2019 Jul 29;11(8):1747. doi: 10.3390/nu11081747 (PMC6723101; doi:10.3390/nu11081747)
Supplement: Supplementary file 1 [file nutrients-11-01747-s001.pdf]

Table S1. Lifestyle habits of participants grouped on the basis of BMI categories.

Parameters	Normal weight n=79; 17.3%	Over weight n=89; 19.5%	Grade I obesity n=86; 18.9%	Grade II obesity n=91; 20.0%	Grade III obesity n=110; 24.1%	*p*-value
Age (years)	36±11.2	35±11.2	37±12.5	36±11.1	36±9.9	0.88
Smoking (yes)						
Yes	28, 35.4%	26, 29.2%	30, 34.9%	25, 27.5%	37, 33.6%	χ^2=2.1
No	51, 64.6%	63, 70.8%	56, 65.1%	66, 72.5%	73, 66.4%	*p*=0.72
Physical activity (yes)						
Yes	16, 20.3%	24, 27.0%	31, 36.0%	25, 27.5%	22, 20.0%	χ^2=8.1
No	63, 79.7%	65, 73.0%	55, 64.0%	66, 72.5%	88, 80.0%	*p*=0.09

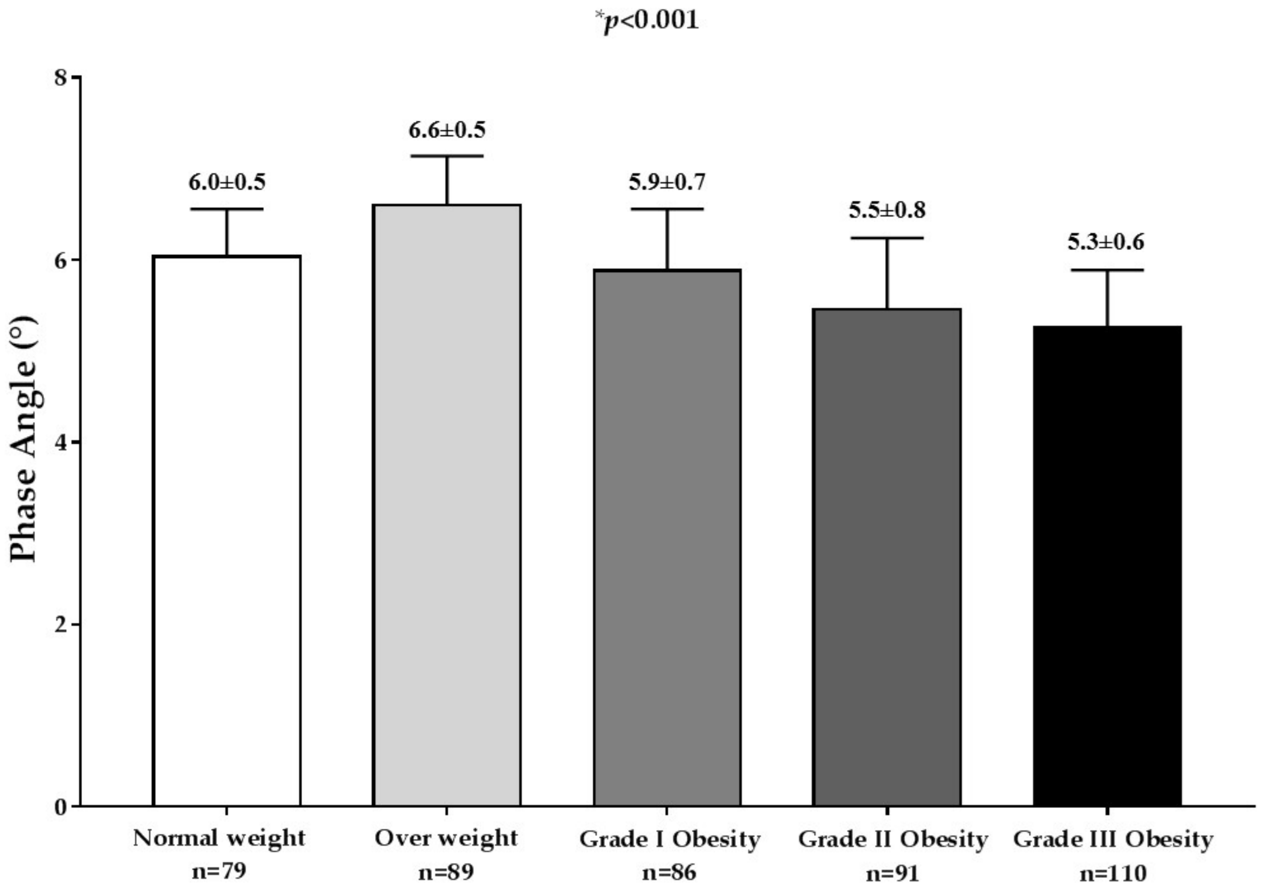

Figure S1. The difference of PhA across all body mass index (BMI) categories. A *p* value in bold type denotes a significant difference (*$p < 0.05$).
